# Non-Hodgkin Lymphoma Risk and Insecticide, Fungicide and Fumigant Use in the Agricultural Health Study

**DOI:** 10.1371/journal.pone.0109332

**Published:** 2014-10-22

**Authors:** Michael C. R. Alavanja, Jonathan N. Hofmann, Charles F. Lynch, Cynthia J. Hines, Kathryn H. Barry, Joseph Barker, Dennis W. Buckman, Kent Thomas, Dale P. Sandler, Jane A. Hoppin, Stella Koutros, Gabriella Andreotti, Jay H. Lubin, Aaron Blair, Laura E. Beane Freeman

**Affiliations:** 1 Division of Cancer Epidemiology and Genetics, National Cancer Institute, Rockville, Maryland, United States of America; 2 College of Public Health, University of Iowa, Iowa City, Iowa, United States of America; 3 National Institute for Occupational Safety and Health, Cincinnati, Ohio, United States of America; 4 IMS, Inc, Calverton, Maryland, United States of America; 5 National Exposure Research Laboratory, U.S. Environmental Protection Agency, Research Triangle Park, North Carolina, United States of America; 6 Epidemiology Branch, National Institute for Environmental Health Sciences, Research Triangle Park, North Carolina, United States of America; Kagoshima University Graduate School of Medical and Dental Sciences, Japan

## Abstract

Farming and pesticide use have previously been linked to non-Hodgkin lymphoma (NHL), chronic lymphocytic leukemia (CLL) and multiple myeloma (MM). We evaluated agricultural use of specific insecticides, fungicides, and fumigants and risk of NHL and NHL-subtypes (including CLL and MM) in a U.S.-based prospective cohort of farmers and commercial pesticide applicators. A total of 523 cases occurred among 54,306 pesticide applicators from enrollment (1993–97) through December 31, 2011 in Iowa, and December 31, 2010 in North Carolina. Information on pesticide use, other agricultural exposures and other factors was obtained from questionnaires at enrollment and at follow-up approximately five years later (1999–2005). Information from questionnaires, monitoring, and the literature were used to create lifetime-days and intensity-weighted lifetime days of pesticide use, taking into account exposure-modifying factors. Poisson and polytomous models were used to calculate relative risks (RR) and 95% confidence intervals (CI) to evaluate associations between 26 pesticides and NHL and five NHL-subtypes, while adjusting for potential confounding factors. For total NHL, statistically significant positive exposure-response trends were seen with lindane and DDT. Terbufos was associated with total NHL in ever/never comparisons only. In subtype analyses, terbufos and DDT were associated with small cell lymphoma/chronic lymphocytic leukemia/marginal cell lymphoma, lindane and diazinon with follicular lymphoma, and permethrin with MM. However, tests of homogeneity did not show significant differences in exposure-response among NHL-subtypes for any pesticide. Because 26 pesticides were evaluated for their association with NHL and its subtypes, some chance finding could have occurred. Our results showed pesticides from different chemical and functional classes were associated with an excess risk of NHL and NHL subtypes, but not all members of any single class of pesticides were associated with an elevated risk of NHL or NHL subtypes. These findings are among the first to suggest links between DDT, lindane, permethrin, diazinon and terbufos with NHL subtypes.

## Introduction

Since the 1970s, epidemiologic studies of non-Hodgkin lymphoma (NHL) and multiple myeloma (MM) have shown increased risk among farmers and associations with the type of farming practiced [1]–[6]. While farmers are exposed to many agents that may be carcinogenic [7]; there has been a particular focus on pesticides. Studies from around the world have suggested increased risk of NHL or MM [8], [9] and other NHL subtypes [10] in relation to the use of specific pesticides in different functional classes (i.e., insecticides, fungicides, fumigants and herbicides). A meta-analysis of 13 case-control studies published between 1993–2005 observed an overall significant meta-odds ratio (OR) between occupational exposure to pesticides and NHL (OR = 1.35; 95% CI: 1.2–1.5) [11]. This risk was greater among individuals with more than 10 years of exposure (OR = 1.65; 95% CI: 1.08–1.95) [11], but the meta-analysis lacked details about the use of specific pesticides and other risk factors [11]. Although the International Agency for Research on Cancer (IARC) has classified “Occupational exposures in spraying and application of non-arsenical insecticides” as “probably carcinogenic to humans”, the human evidence for the 17 individual pesticides evaluated in this monograph was determined to be inadequate for nine and there were no epidemiological studies for eight pesticides [12]. Since then, more studies have focused on cancer risk from specific pesticides, although the information is still relatively limited for many cancer-pesticide combinations [8], [9].

To help fill the current information gap we evaluated the relationships between the use of specific insecticides, fungicides and fumigants and NHL in the Agricultural Health Study (AHS), a prospective cohort of licensed private (i.e., mostly farmer) and commercial pesticide applicators. Because the etiology of NHL and its B and T cell subtypes may differ by cell type^13^, we also evaluated risk by subtype while controlling for potential confounding factors suggested from the literature [13], and the AHS data.

### Novelty and Impact

These findings on occupationally exposed pesticide applicators with high quality exposure information are among the first to suggest links between DDT, lindane, permethrin, diazinon and terbufos and specific NHL subtypes in a prospective cohort study.

## Materials and Methods

### Study Population

The AHS is a prospective cohort study of 52,394 licensed private pesticide applicators (mostly farmers) in Iowa and North Carolina and 4,916 licensed commercial applicators in Iowa (individuals paid to apply pesticides to farms, homes, lawns, etc.), and 32,346 spouses of private applicators. Only applicators are included in this analysis. The cohort has been previously described in detail [14], [15] and study questionnaires are available on the AHS website (www.aghealth.nih.gov). Briefly, individuals seeking licenses to apply restricted use pesticides were enrolled in the study from December 1993 through December 1997 (82% of the target population enrolled). At enrollment, subjects did not sign a written informed consent form. However, the cover letter of the questionnaire booklet informed subjects of the voluntary nature of participation, the ability to not answer any question, and it provided an assurance of confidentiality (including a Privacy Act Notification statement). The letter also included a written summary of the purpose of research, time involved, benefits of research, and a contact for questions about the research. The cover letter to the take-home questionnaire included all of the above and also informed the participant that they had the right to withdraw at any time. Finally, subjects were specifically informed that their contact information (including Social Security Number) would be used to search health and vital records in the future. The participants provided consent by completing and returning the questionnaire booklet. These documents and procedures were approved in 1993 by all relevant institutional review boards (i.e., National Cancer Institute Special Studies Institutional Review Board, Westat Institutional Review Board, and the University of Iowa Institutional Review Board-01).

Excluded from this analysis were study participants who had a history of any cancer at the time of enrollment (n = 1094), individuals who sought pesticide registration in Iowa or North Carolina but did not live in these states at the time of registration (n = 341) and were thus outside the catchment area of these cancer registries and individuals that were missing information on potential confounders (i.e., race or total herbicides application days [n = 1,569]). This resulted in an analysis sample of 54,306. We obtained cancer incidence information by regular linkage to the population-based cancer registry files in Iowa and North Carolina. In addition, we linked cohort members to state mortality registries of Iowa and North Carolina and the nation-wide National Death Index to determine vital status, and to the nation-wide address records of the Internal Revenue Service, state-wide motor vehicle registration files, and pesticide license registries of state agricultural departments to determine residence in Iowa or North Carolina. The current analysis included all incident primary NHL, as well as CLL and MM (which are now classified as NHL) [13] (*n* = 523) diagnosed from enrollment (1993–1997) through December 31, 2010 in North Carolina and from enrollment (1993–1997) through December 31, 2011 in Iowa, the last date of complete cancer incidence reports in each state. We ended follow-up and person-year accumulation at the date of diagnosis of any cancer, death, movement out of state, or December 31, 2010 in North Carolina and December 31, 2011 in Iowa, whichever was earlier.

### Tumor Characteristics

Information on tumor characteristics was obtained from state cancer registries. We followed the definition of NHL and six subtypes of NHL used by the Surveillance Epidemiology and End Results (SEER) coding scheme [16] which was based on the Pathology Working Group of the International Lymphoma Epidemiology Consortium *(*ICD-O-3 InterLymph modification) classification (Table S1 in File S1, [17], i.e., 1. Small B-cell lymphocytic lymphomas (SLL)/chronic B-cell lymphocytic lymphomas (CLL)/mantle-cell lymphomas (MCL); 2. Diffuse large B-cell lymphomas; 3. Follicular lymphomas; 4. ‘Other B-cell lymphomas’ consisting of a diverse set of B-cell lymphomas; 5. Multiple myeloma; and 6. T-cell NHL and undefined cell type). There were too few T-cell NHL cases available for analysis [n = 19] so this cell type was not included in the subtype analysis). The ICD-O-3 original definition (used in many earlier studies of pesticides and cancer) of NHL [18] was also evaluated in relation to pesticide exposure to allow a clearer comparison of our results with previous studies.

### Exposure Assessment

Initial information on lifetime use of 50 specific pesticides (Table S2 in File S1), including 22 insecticides, 6 fungicides and 4 fumigants was obtained from two self-administered questionnaires [14], [15] completed during cohort enrollment (Phase 1). All 57,310 applicators completed the first enrollment questionnaire, which inquired about ever/never use of 50 pesticides, as well as duration (years) and frequency (average days/year) of use for a subset of 22 pesticides including 9 insecticides, 2 fungicides and 1 fumigant. In addition, 25,291 (44%) of the applicators returned the second (take-home) questionnaire, which inquired about duration and frequency of use for the remaining 28 pesticides, including 13 insecticides, 4 fungicides and 3 fumigants.

A follow-up questionnaire, which ascertained pesticide use since enrollment, was administered approximately 5 years after enrollment (1999–2005, Phase 2) and completed by 36,342 (63%) of the original participants. The full text of the questionnaires is available at www.aghealth.nih.gov. For participants who did not complete the Phase 2 questionnaire (20,968 applicators, 37%), a data-driven multiple imputation procedure which used logistic regression and stratified sampling [19] was employed to impute use of specific pesticides in Phase 2. Information on pesticide use from Phase 1, Phase 2 and imputation for Phase 2 was used to construct three cumulative exposure metrics: (i) lifetime days of pesticide use (i.e., the product of years of use of a specific pesticide and the number of days used per year); (ii) intensity-weighted lifetime days of use (i.e., the product of lifetime days of use and a measure of exposure intensity) and (iii) ever/never use data for each pesticide. Intensity was derived from an exposure-algorithm, which was based on exposure measurements from the literature and individual information on pesticide use and practices (e.g., whether or not they mixed pesticides, application method, whether or not they repaired equipment and use of personal protective equipment) obtained from questionnaires completed by study participants [20].

### Statistical Analyses

We divided follow-up time into 2-year intervals to accumulate person-time and update time-varying factors, such as attained age and pesticide use. We fit Poisson models to estimate rate ratios (RRs) and 95% confidence intervals (95% CI) to evaluate the effects of pesticide use on rates of overall NHL and the five NHL subtypes.

We evaluated pesticides with 15 or more exposed cases of total NHL, thereby excluding aluminum phosphide, carbon tetrachloride/carbon disulfide, ethylene dibromide, trichlorfon, and ziram leaving 26 insecticides, fungicides and fumigants for analysis (permethrin for animal use and crop use were combined into one category, all insecticides, fungicides and fumigants are listed in Table S2 in File S1). For each pesticide, we evaluated ever vs. never exposure, as well as tertiles of exposure which were created based on the distribution of all NHL exposed cases and compared to those unexposed. In the NHL subtype analysis and in circumstances where multiple pesticides were included in the model we categorized exposure for each pesticide into unexposed (i.e., never users) and two exposed groups (i.e., low and high) separated at the median exposure level. The number of exposed cases included in the ever/never analysis and in the trend analysis can differ because of the lack of information necessary to construct quantitative exposure metrics for some individuals.

Several lifestyle and demographic factors associated with NHL in the AHS cohort or previously suggested as possible confounders in the NHL literature^13^ were evaluated as potential confounders in this analysis. These included: age at enrollment, gender, race, state, license type, education, autoimmune diseases, family history of lymphoma in first-degree relatives, body mass index, height, cigarette smoking history, alcohol consumption per week and several occupational exposures^1–13^ including number of livestock, cattle, poultry, whether they raised poultry, hogs or sheep, whether they provided veterinary services to their animals, number of acres planted, welding, diesel engine use, number of years lived on the farm, total days of any pesticide use, and total days of herbicide use. However, since most of these variables did not change the risk estimates for specific pesticides, we present results adjusted for age, race, state and total days of herbicide use, which impacted risk estimates by more than 10% for some subtypes. We also performed analyses adjusting for specific insecticides, fungicides and fumigants shown to be associated with NHL or a specific NHL subtype in the current analysis. Tests for trend used the median value of each exposure category. All tests were two-sided and conducted at α = 0.05 level. Analysis by NHL subtype was limited to insecticides, fungicides, and fumigants with 6 or more exposed cases.

We also fit polytomous logit models, where the dependent variable was a five-level variable (i.e., five NHL subtypes) and a baseline level (i.e., no NHL) to estimate exposure-response odds ratios (ORs) and 95% confidence intervals (CIs) for each subtypes of NHL. We then used polytomous logit models to estimate exposure-response trend while adjusting for age, state, race and total days of herbicide use, as in the Poisson models, and tested homogeneity among the 5 NHL subtypes.

Poisson models were fit using the GENMOD procedure and polytomous logit models were fit using the LOGISTIC procedure of the SAS 9.2 statistical software package (SAS Institute, Cary, NC). Summary estimates of NHL and NHL subtype risks for both Poisson models and polytomous logit models incorporated imputed data and were calculated along with standard error estimates, confidence intervals, and p-values, using multiple imputation methods implemented in the MIANALYZE procedure of SAS 9.2.

We also evaluated the impact of the additional pesticide exposure information imputed for Phase 2 on risk estimates. We compared risk estimates for those who completed both the phase 1 enrollment and take-home questionnaires and the phase 2 questionnaires (n = 17,545) with risk estimates obtained from the combined completed questionnaire data plus the imputed phase 2 data (n = 54,306). We also explored the effect of lagging exposure data 5 years because recent exposures may not have had time to have an impact on cancer development. For comparison to previous studies, we also assessed the exposure-response association for NHL using the original ICD-O-3 definition of NHL [18] and the new definition [16] in Table S3 in File S1. Unless otherwise specified, reported results show un-lagged exposure information from both Phase 1 and Phase 2 including Phase 2 imputed data for lifetime exposure-days and intensity-weighted lifetime days of use and NHL defined by the InterLymph modification of ICD-O-3 [17]. Data were obtained from AHS data release versions P1REL201005.00 (for Phase 1) and P2REL201007.00 (for Phase 2).

## Results

The 54,306 applicators in this analysis contributed 803,140 person-years of follow-up from enrollment through December 31, 2010 in North Carolina and December 31, 2011 in Iowa (Table 1). During this period, there were 523 incident cases of NHL, including 148 SLL/CLL/MCL, 117 diffuse large B-cell lymphomas, 67 follicular lymphomas, 53 ‘other B-cell lymphomas’ (consisting of a diverse set of B-cell lymphomas) and 97 cases of MM. Another 41 cases consisting of T-cell lymphomas (n = 19) and non-Hodgkin lymphoma of unknown lineage (n = 22) were excluded from cell type-specific analyses because of small numbers of cases with identified cell types. Between enrollment and the end of follow-up, 6,195 individuals were diagnosed with an incident cancer other than NHL, 4,619 died without a record of cancer in the registry data, and 1,248 cohort members left the state and could not be followed-up for cancer. Person-years of follow-up accumulated for all of these study participants after enrollment until they were censored for the incident cancer, death or moving out of the state (data not shown). The risk of NHL increased significantly and monotonically with age in the AHS cohort in this analysis (p = 0.001) and age-adjusted risks were significant for state and NHL overall and race for multiple myeloma (data not shown). Total days of herbicide use had a small but significant effect on the risk of some NHL subtypes, but not on NHL overall. No other demographic or occupational factors showed evidence of confounding so they were not included in the final models.

**Table 1:** Baseline characteristics of AHS study participants in the NHL incidence analysis^1^^,^ 2.

Variables	All NHL cases (%)	Cohort Person-years.
**Age at Enrollment**		
<45	84 (16.1)	426,288
45–49	51 (9.8)	101,018
50–54	75 (14.3)	84,998
55–59	90 17.2)	74,440
60–64	78 (14.9)	56,978
65–69	79 (15.1)	35,071
≥70	66 (12.6)	24,347
**Race**		
White	509 (97.3)	787,799
Black	14 (2.7)	15,341
**State**		
IA	332 (63.5)	537,252
NC	191 (36.5)	265,888
**Lifetime Total Herbicide Exposure Days**		
0–146 days	170 (32.5)	251,401
147–543 days	169 (32.3)	273,107
544–2453 days	184 (35.2)	278,632

1 During the period from enrollment (1993–1997) to December 31, 2010 in NC and December 31, 2011 in Iowa.

2 Individuals with missing ever/never exposure information or missing confounding variable information were not included in the table.

In Table 2 we present ever/never results for 26 insecticides, fungicides and fumigants by total NHL and by NHL subtype adjusted for age, race, state and herbicide use (total life-time days). Terbufos was the only pesticide associated with an increased risk of total NHL in the ever/never use analysis (RR = 1.2 [1.0–1.5]), although the trend for increasing use and risk of total NHL was not significant (p trend = 0.43) (Table 3). In contrast, there were a few chemicals that were not associated with ever/never use, but did show evidence of an exposure-response association. Lindane was the only pesticide that showed a statistically significant increasing trend in risk for NHL with both exposure metrics, for lifetime-days of lindane use the RR were = 1.0 (ref), 1.2 (0.7–1.9), 1.0 (0.6–1.7), 2.5 (1.4–4.4); p trend = 0.004 and intensity-weighted lifetime-days of use the: RR were:  = 1.0 (ref), 1.3 (0.8–2.2), 1.1 (0.7–1.8), 1.8 (1.0–3.2); p trend = 0.04. DDT showed a significant trend for NHL risk with life-time days of use RR = 1.0 (ref), 1.3 (0.9–1.8), 1.1 (0.7–1.7), 1.7 (1.1–2.6); p trend = 0.02, while the intensity weighted lifetime days of use of DDT was of borderline significance: RR = 1.0 (ref), 1.2 (0.8–1.8), 1.1 (0.8–1.7), 1.6 (1.0–2.3); p trend = 0.06. The number of lifetime days of use of lindane and DDT was weakly correlated (coefficient of determination = 0.04), and the pattern of NHL risk showed little change when both were included in the model. The results for lindane adjusted for DDT were, RR = 1.0 (ref), 1.2 (0.7–2.0), 1.0 (0.5–1.8), 1.6 (0.9–3.3); p trend = 0.07 and the results for DDT adjusted for lindane were, RR = 1.0 (ref), 1.3 (0.9–2.0), 0.9 (0.6–1.6), 1.6 (0.9–2.6); p trend = 0.08).

**Table 2:** Pesticides exposure (ever/never) and adjusted Relative Risk of total NHL and NHL Subtype^1^.

Insecticide													
	Total NHL Cases^2^		SLL/CLL/MCL Cases^2^		Diffuse Large B-CellCases^2^		Follicular B-CellCases^2^		Other B-cell Cases^2^		Multiple Myeloma Cases^2^		
Pesticide(chemical-functional class)	Ever/NeverExposed	RR^3^^,^ 4	Ever/NeverExposed	RR^3^^,^ 4	Ever/NeverExposed	RR^3^^,^ 4	Ever/NeverExposed	RR^3^^,^ 4	Ever/NeverExposed	RR^3^^,^ 4	Ever/NeverExposed	RR^3^^,^ 4	
		(95% CI)		(95% CI)		(95% CI)		(95% CI)		(95% CI)		(95% CI)	
**Aldicarb**	47/435	1	14/124	1.1	8/98	0.7	6/54	0.9	7/41	1.6	10/82	1.2	
**(carbamate-insecticide)**		(0.7–1.4)		(0.6–1.8)		(0.4–1.5)		(0.3–2.2)		(0.7–3.5)		(0.6–2.2)	
**Carbofuran**	147/317	1.1	48/86	1.2	26/78	0.8	18/39	1	13/31	0.8	31/56	1.3	
**(carbamate-insecticide)**		(0.9–1.3)		(0.8–1.8)		(0.5–1.3)		(0.5–1.7)		(0.4–1.6)		(0.8–2.1)	
**Carbaryl**	272/225	1	75/66	1	58/53	0.8	37/24	0.8	24/28	0.9	58/34	0.9	
**(carbamate-insecticide)**		(0.8–1.2)		(0.7–1.5)		(0.5–1.3)		(0.5–1.3)		(0.5–1.6)		(0.6–1.4)	
**Chlorpyrifos**	210/300	1	62/84	1	44/70	0.9	32/33	1.3	21/31	0.8	36/58	1	
**(organophosphate-insecticide)**		(0.8–1.2)		(0.7–1.4)		(0.6–1.4)		(0.8–2.2)		(0.5–1.5)		(0.6–1.5)	
**Coumaphos**	46/411	1.1	15/120	1.2	10/93	1	8/48	1.6	5/40	xxx	7/78	1	
**(organophos-phate-insecticide)**		(0.8–1.5)		(0.7–2.1)		(0.5–2.1)		(0.8–3.5)				(0.1–2.1)	
**DDVP**	55/407	1.1	13/124	0.8	10/93	1	8/48	1.3	6/39	1	12/73	1.7	
**(dimethyl phosphate-insecticide)**		(0.8–1.5)		(0.5–1.5)		(0.5–1.9)		(0.6–2.7)		(0.4–2.4)		(0.9–3.2)	
**Diazinon**	144/342	1	46/93	1.3	30/78	0.9	22/38	1.3	12/37	0.8	27/64	1	
**(organophosphorous-insecticide)**		(0.8–1.3)		(0.9–1.9)		(0.6–1.4)		(0.7–2.3)		(0.4–1.6)		(0.6–1.6)	
**Fonofos**	115/349	1.1	35/100	1.1	25/81	1.2	13/45	0.9	15/30	1.3	19/66	1.3	
**(organophosphorous-insecticide)**		(0.9–1.4)		(0.7–1.6)		(0.7–1.9)		(0.5–1.7)		(0.7–2.5)		(0.8–2.3)	
**Malathion**	332/163	0.9	99/43	1	72/37	0.9	46/14	1.3	30/21	0.6	61/32	0.9	
**(organophosphorous-insecticide)**		(0.8–1.1)		(0.7–1.4)		(0.6–1.4)		(0.7–2.4)		(0.3–1.0)		(0.6–1.5)	
**Parathion (ethyl or methyl)**	69/411	1.1	20/117	1	14/91	1	10/48	1.1	7/44	1.1	14/77	1	
**(organophosphorous insecticide**		(0.8–1.4)		(0.7–1.4)		(0.6–1.4)		(0.8–1.5)		(0.7–1.5)		(0.8–1.5)	
**Permethrin** **(animal and crop applications)**	112/363	1.1	32/106	1	18/81	0.7	18/81	1.1	9/14	0.8	20/72	**2.2**	
**(pyrethroid insecticide)**		(0.8–1.3)		(0.6–1.5)		(0.4–1.2)		(0.6–2.0)		(0.4–1.6)		**(1.4–3.5)**	
**Phorate**	160/325	1	53/87	1.1	31/76	0.9	20/40	0.9	19/31	0.9	26/64	1	
**(organophosphorous-insecticide)**		(0.8–1.2)		(0.8–1.6)		(0.5–1.3)		(0.5–1.6)		(0.5–1.6)		(0.6–1.7)	
**Terbufos**	201/267	**1.2**	64/72	1.4	42/63	1.1	31/26	1.2	26/19	1.8	32/59	1.2	
**(organophosphorous-insecticide)**		**(1.0–1.5)**		(0.97–2.0)		(0.7–1.7)		(0.7–2.1)		(0.94–3.2)		(0.7–1.9)	
**Chlorinated Insecticides**													
**Aldrin**	116/364	0.9	53/99	0.9	15/91	0.8	13/45	0.8	12/37	0.6	29/62	1.5	
**(chlorinated insecticide)**		(0.7–1.1)		(0.6–1.4)		(0.4–1.6)		(0.4–1.6)		(0.3–1.3)		(0.9–2.5)	
**Chlordane**	136/344	1	49/90	1.4	20/86	0.6	18/41	1.2	13/36	1	31/60	1.2	
**(chlorinated insecticide)**		(0.8–1.3)		(0.99–2.1)		(0.4–1.0)		(0.7–2.1)		(0.7–2.0)		(0.8–1.9)	
**DDT**	182/300	1	59/79	1.2	34/73	0.8	18/41	0.9	20/31	1.1	40/50	1.1	
**(chlorinated insecticide)**		(0.8–1.3)		(0.8–1.8)		(0.5–1.3)		(0.5–1.6)		(0.6–2.1)		(0.7–1.8)	
**Dieldrin**	35/442	0.9	5/130	xxx	4/101	xxx	4/54	xxx	7/42	1	10/81	0.9	
**(chlorinated insecticide)**		(0.6–1.2)								(0.7–2.0)		(0.5–1.4)	
**Heptachlor**	90/384	1	33/104	1.1	10/95	1.1	9/48	1.1	13/36	0.9	17/72	1.1	
**(chlorinated insecticide)**		(0.7–1.2)		(0.7–3.0)		(0.3–3.1)		(0.5–3.2)		(0.5–2.7))		(0.6–2.0)	
**Lindane**	85/396	1	27/113	1.2	12/95	0.6	16/41	1.7	9/40	0.7	13/73	1.1	
**(chlorinated insecticide)**		(0.8–1.2)		(0.6–1.5)		(0.3–1.1)		(0.96–3.2)		(0.4–1.2)		(0.5–2.0)	
**Toxaphene**	79/397	1	21/116	0.9	14/90	0.8	9/47	1	10/40	1.1	19/73	1.1	
**(chlorinated insecticide)**		(0.7–1.2)		(0.5–1.5)		(0.4–1.4)		(0.6–2.0)		(0.6–2.0)		(0.6–1.9)	
**Fungicides**													
**Benomyl**	54/428	1.1	18/123	1.2	12/95	1.1	4/51	xxx	4/51	xxx	11/80	1.1	
**(carbamate fungicide)**		(0.8–1.5)		(0.7–2.0)		(0.6–1.9)						(0.6–2.0)	
**Captan**	60/406	1.1	18/118	1.1	12/91	0.9	5/51	xxx	6/39	1.1	12/76	1.2	
**(phthalimide fungicide)**		(0.8–1.4)		(0.6–1.8)		(0.5–1.8)				(0.5–2.7)		(0.6–2.2)	
**Chloro-thalonil**	35/474	0.8	9/135	0.9	6/107	0.5	5/60	xxx	2/50	xxx	11/84	1.2	
**(poly-chlorinated aromatic** **thalonitrile fungicide)**		(0.5–1.2)		(0.4–1.9)		(0.2–1.3)						(0.6–2.3)	
**Maneb/**	44/437	0.9	13/127	1.1	12/95	1.1	4/60	xxx	5/49	xxx	10/79	0.8	
**Mancozeb**		(0.7–1.3)		(0.6–2.1)		(0.6–2.1)						(0.4–1.7)	
**(dithiocarbamate fungicide)**													
**Metalaxyl**	108/381	1	34/106	**1.6**	27/82	1.1	10/48	0.7	10/40	0.9	21/71	0.8	
**(acylalanine fungicide)**		(0.8–1.3)		**(1.0–2.5)**		(0.6–1.8)		(0.4–1.4)		(0.4–1.7)		(0.4–1.3)	
**Fumigant**													
**Methyl bromide**	85/425	1.1	18/126	0.9	28/86	**1.9**	7/58	0.6	8/44	2.2	19/76	1	
**(methyl halide fumigant)**		(0.9–1.5)		(0.5–1.7)		**(1.1–3.3)**		(0.2–1.4)		(0.9–5.7)		(0.6–1.8)	

1 During the period from enrollment (1993–1997) to December 31, 2010 in NC and December 31, 2011 in Iowa.

2 Numbers of cases by NHL subtype do not sum to total number of NHL cases (n = 523) due to missing data.

3 Adjusted RR: age (<45, 45–49, 50–54, 55–59, 60–64, 65–69, ≥70), State (NC vs. IA), Race (White vs. Black), AHS herbicides (tertiles of total herbicide use-days). Statistically significant RR and 95% confidence limits are bolded.

4 RR was not calculated if the number of exposed cases in a pesticide-NHL subtype cell was <6 and the missing RR was marked with an XXX. Statistically significant RRs and 95% confidence limits are bolded.

**Table 3:** Pesticide exposure (lifetime-days & intensity weighted life-time days) and adjusted risks of total NHL incidence^1^.

Insecticides						
Pesticide(chemical-functional class)	NHL Cases^2^	Non-Cases^2^	RR^3^^,^ 4 (95% CI) byTotal Days of Exposure	NHL	Non-Cases	RR^3^^,^ 4 (95% CI)
[days of lifetime exposure foreach category]				Cases^2^^,^		Intensity-weighteddays of exposure
**Aldicarb** **(carbamate-insecticide)**						
None	238	21557	1.0 (ref)	238	21557	1.0 (ref)
Low [≤8.75]	7	633	1.1 (0.5–2.3)	6	383	1.3 (0.6–3.3))
Medium [>8.75–25.5]	5	522	0.9 (0.3–2.5)	6	853	0.9 (0.4–1.9)
High [>25.5–224.75]	5	1266	0.5 (0.2–1.3)	5	1183	0.5 (0.2–1.3)
			P trend = 0.23			P trend = 0.22
**Carbofuran** **(carbamate-insecticide)**						
None	317	36296	1.0 (ref)	317	36296	1.0 (ref)
Low [≤8.75]	63	4775	1.2 (0.9–1.6)	46	3695	1.2 (0.9–1.6)
Medium [>8.75–38.75]	32	3648	0.8 (0.6–1.2)	46	4590	1.0 (0.7–1.3)
High [>38.75–767.25]	44	4370	0.97 (0.7–1.4)	45	4477	1.0 (0.7–1.4)
			P trend = 0.69			P trend = 0.74
**Carbaryl** **(carbamate-insecticide)**						
None	128	12864	1.0 (ref)	128	12864	1.0 (ref)
Low [≤8.75]	54	4128	1.1 (0.7–1.6)	46	3962	1.0 (0.7–1.5)
Medium [8.75–56]	43	5096	0.9 (0.6–1.2)	45	4433	0.9 (0.7–1.5)
High [>56–737.5]	39	3281	1.0 (0.7–1.6)	44	4029	1.0 (0.6–1.5)
			P trend = 0.87			P trend = 0.94
**Chlorpyrifos** **(organophosphate-insecticide)**						
None	300	30393	1.0 (ref)	300	30393	1.0 (ref)
Low [≤8.75]	71	6493	1.1 (0.9–1.5)	61	6383	1.1 (0.8–1.4)
Medium [>8.75–44]	65	6892	1.1 (0.8–1.4)	60	7549	0.9 (0.7–1.2)
High [>44–767.25]	67	9380	0.8 (0.6–1.1)	60	7044	1.0 (0.7–1.3)
			P trend = 0.11			P trend = 0.85
**Coumaphos** **(organophosphate-insecticide)**						
None	411	44846	1.0 (ref)	411	44846	1.0 (ref)
Low [<8.75]	16	1510	1.0 (0.6–1.7)	15	1132	1.3 (0.8–2.1)
Medium [>8.75–38.75]	14	1076	1.2 (0.7–2.1)	14	1452	1.0 (0.6–1.6)
High [>38.75–1627.5]	13	1175	1.2 (0.7–2.0)	14	1170	1.2 (0.7–2.1)
			P for trend = 0.50			P trend = 0.48
**DDVP** **(dimethyl phosphate-insecticide)**						
None	407	44551	1.0 (ref)	407	44551	1.0 (ref)
Low [≤8.75]	19	1342	1.4 (0.9–2.1)	18	1281	1.4 (0.9–2.3)
Medium [>8.75–87.5]	17	1519	1.2 (0.7–1.9)	18	1633	1.1 (0.7–1.8)
High [>87.5–2677.5]	17	1893	0.9 (0.6–1.5)	17	1824	1.0 (0.6–1.6)
			P trend = 0.78			P trend = 0.83
**Diazinon** **(organophosphorous-insecticide)**						
None	187	17943	1.0 (ref)	187	17943	1.0 (ref)
Low [≤8.75]	28	2506	1.1 (0.7–1.6)	23	2047	1.1 (0.7–1.8)
Medium [>8.75–25]	19	1515	1.0 (0.6–1.8)	24	2246	0.9 (0.5–1.5)
High [>25–457.25]	23	1990	1.2 (0.7–1.9)	22	1708	1.3 (0.8–2.1)
			P trend = 0.52			P trend = 0.33
**Fonofos** **(organophosphorous-insecticide)**						
None	349	39570	1.0 (ref)	349	39570	1.0 (ref)
Low [≤20]	47	3812	1.3 (0.96–1.8)	37	2906	1.4 (0.97–1.9)
Medium [>20–50.75]	28	2819	1.1 (0.7–1.6)	38	3487	1.1 (0.8–1.6)
High [>50.75–369.75]	37	3385	1.1 (0.7–1.5)	36	3606	1.0 (0.7–1.4)
			P trend = 0.83			P trend = 0.87
**Malathion** **(organophosphorous-insecticide)**						
None	90	8368	1.0 (ref)	90	8368	1.0 (ref)
Low [≤8.75]	75	7284	0.97 (0.7–1.3)	60	5535	1.0 (0.7–1.4)
Medium [>8.75–38.75]	47	5779	0.7 (0.5–1.1)	59	6899	0.8 (0.6–1.1)
High [>38.75–737.5]	57	5037	0.9 (0.6–1.3)	59	5588	0.9 (0.6–1.2)
			P trend = 0.63			P trend = 0.46
**Parathion (ethyl or methyl)** **(organophosphorous insecticide)**						
None	228	21457	1.0 (ref)	228	21457	1.0 (ref)
Low [≤8.75]	9	693	1.0 (0.5–2.0)	7	612	0.9 (0.4–2.0)
Medium [>8.75–24.5]	6	351	1.4 (0.6–3.2)	8	462	1.4 (0.7–2.9)
High [>.24.5–1237.5]	6	652	0.8 (0.3–1.8)	6	621	0.8 (0.4–1.9)
			P trend = 0.64			P trend = 0.74
**Permethrin** **(animal and crop applications)**						
**(pyrethroid insecticide)**						
None	371	37496	1.0 (ref)	371	37496	1.0 (ref)
Low [≤8.75]	38	4315	1.1 (0.8–1.5)	33	4263	0.9 (0.6–1.3)
Medium [>8.75–50.75]	31	4611	0.8 (0.5–1.2)	33	4200	1.0 (0.7–1.4)
High [>50.75–1262.25]	33	4121	1.2 (0.8–1.7)	32	4553	1.0 (0.7–1.5)
			P trend = 0.54			P trend = 0.99
**Phorate** **(organophosphorous-insecticide)**						
None	171	16834	1.0 (ref)	171	16834	1.0 (ref)
Low [≤8.75]	27	2621	0.8 (0.5–1.2)	26	2320	0.9 (0.6–1.4)
Medium [8.75–24.5]	33	1819	1.4 (0.96–2.1)	27	1951	1.1 (0.7–1.7)
High [>24.5–224.75]	18	2246	0.6 (0.4–1.1)	25	2409	0.8 (0.5–1.3)
			P trend = 0.25			P trend = 0.44
**Terbufos** **(organophosphorous-insecticide)**						
None	267	31076	1.0 (ref)	267	31076	1.0 (ref)
Low [≤24.5]	82	8410	1.2 (0.9–1.5)	64	6895	1.1 (0.9–1.5)
Medium [>24.5–56]	54	3925	1.6 (1.2–2.1)	64	4642	1.6 (1.2–2.2)
High [>56–1627.5]	57	6080	1.1 (0.8–1.5)	63	6842	1.1 (0.8–1.5)
			P trend = 0.43			P trend = 0.44
**Chlorinated Insecticides**						
**Aldrin** **(chlorinated insecticide)**						
None	193	19743	1.0 (ref)	193	19743	1.0 (ref)
Low [≤8.75]	27	1613	0.9 (0.6–1.4)	20	1212	0.9 (0.6–1.4)
Medium [>8.75–24.5]	16	1002	0.8 (0.5–1.3)	20	1279	0.8 (0.5–1.3)
High [>24.5–457.25]	17	903	0.9 (0.5–1.5)	19	1026	0.9 (0.6–1.5)
			P trend = 0.58			P trend = 0.74
**Chlordane** **(chlorinated insecticide)**						
None	179	19115	1.0 (ref)	179	19115	1.0 (ref)
Low [≤8.75]	47	2687	1.3 (0.97–1.9)	23	1303	1.4 (0.9–2.2)
Medium^5^	0	0	xxx	24	1747	1.0 (0.6–1.5)
High [>8.75–1600]	23	1450	1.1 (0.7–1.7)	22	1085	1.4 (0.9–2.2)
			P trend = 0.43			P trend = 0.16
**DDT** **(chlorinated insecticide)**						
None	152	18543	1.0 (ref)	152	18543	1.0 (ref)
Low [≤8.75]	43	2121	1.3 (0.9–1.8)	33	1601	1.2 (0.8–1.8)
Medium [>8.75–56]	28	1598	1.1 (0.7–1.7)	32	1760	1.1 (0.8–1.7)
High [>56–1627.5]	27	953	1.7 (1.1–2.6)	32	1305	1.6 (1.0–2.3)
			**P trend = 0.02**			P trend = 0.06
**Dieldrin** **(chlorinated insecticide)**						
None	235	22510	1.0 (ref)	235	22510	1.0 (ref)
Low [≤8.75]	7	472	0.7 (0.3–1.5)	6	363	0.8 (0.4–1.8)
Medium [>8.75–24.5]	8	154	2.3 (1.1–4.7)	5	106	2.2 (0.9–5.3)
High [>24.5–224.75]	2	140	0.7 (0.2–2.9)	5	298	0.8 (0.3–2.0)
			P trend = 0.47			P trend = 0.84
**Heptachlor** **(chlorinated insecticide)**						
None	205	20844	1.0 (ref)	205	20844	1.0 (ref)
Low [≤8.75]	21	1261	1.0 (0.6–1.6)	15	1110	0.8 (0.5–1.4)
Medium [>8.75–24.5]	18	679	1.5 (0.9–2.4)	16	425	2.0 (1.2–3.4)
High [>24.5–457.25]	7	600	0.7 (0.3–1.4)	14	1001	0.8 (0.5–1.4)
			P trend = 0.82			P trend = 0.88
**Lindane** **(chlorinated insecticide)**						
None	205	20375	1.0 (ref)	205	20375	1.0 (ref)
Low [≤8.75]	18	1285	1.2 (0.7–1.9)	15	976	1.3 (0.8–2.2)
Medium [>8.75–56]	13	1103	1.0 (0.6–1.7)	16	1205	1.1 (0.7–1.8)
High [>56–457.25]	14	467	2.5 (1.4–4.4)	14	673	1.8 (1.0–3.2)
			**P trend = 0.004**			**P trend = 0.04**
**Toxaphene** **(chlorinated insecticide)**						
None	214	20911	1.0 (ref)	214	20911	1.0 (ref)
Low [≤8.75]	14	1198	0.8 (0.5–1.4)	11	630	1.3 (0.7–2.3)
Medium [>8.75–24.5]	13	564	1.5 (0.9–2.7)	12	931	0.9 (0.5–1.6)
High [>24.5–457.25]	6	686	0.6 (0.3–1.4)	10	886	0.8 (0.4–1.5)
			P trend = 0.50			P trend = 0.38
**Fungicides**						
**Benomyl** **(carbamate fungicide)**						
None	219	21425	1.0 (ref)	219	21425	1.0 (ref)
Low [≤12.25]	14	896	1.7 (0.9–2.9)	9	432	2.2 (1.1–4.3)
Medium [>12.25–24.5]	4	214	2.4 (0.9–6.6)	10	732	1.7 (0.9–3.2)
High [>24.5–457.25]	8	834	1.0 (0.5–2.1)	7	779	0.9 (0.4–2.0)
			P trend = 0.93			P trend = 0.75
**Captan** **(phthalimide fungicide)**						
None	407	43433	1.0 (ref)	407	43433	1.0 (ref)
Low [≤0.25]	15	2334	0.8 (0.5–1.4)	15	2108	0.9 (0.6–1.5)
Medium [>0.25–12.25]	16	1004	1.5 (0.8–2.6)	15	1171	1.2 (0.7–2.2)
High [>12.25–875]	14	1823	0.8 (0.5–1.5)	14	1805	0.8 (0.5–1.5)
			P trend = 0.69			P trend = 0.52
**Chlorothalonil** **(polychlorinated aromatic** **thalonitrile fungicide)**						
None	474	48442	1.0 (ref)	474	48442	1.0 (ref)
Low [≤12.25]	13	1509	0.9 (0.5–1.6)	10	1800	0.6 (0.3–1.2)
Medium [>12.25–64]	9	1492	0.8 (0.4–1.6)	11	1501	0.9 (0.5–1.7)
High [>64–395.25]	9	1678	0.6 (0.3–1.3)	9	1362	0.8 (0.4–1.6)
			P trend = 0.16			PP trend = 0.52
**Maneb/Mancozeb** **(dithiocarbamate fungicide)**						
None	228	21512	1.0 (ref)	228	21512	1.0 (ref)
Low [≤7]	8	400	1.9 (0.9–3.9)	8	486	1.6 (0.8–3.3)
Medium [>7–103.25]	9	990	0.9 (0.4–1.7)	9	680	1.3 (0.6–2.6)
High [>103.25–737.5]	7	454	1.4 (0.6–2.9)	7	677	0.9 (0.4–1.9)
			P trend = 0.49			P trend = 0.78
**Metalaxyl** **(acylalanine fungicide)**						
None	209	18833	1.0 (ref)	209	18833	1.0 (ref)
Low [≤6]	16	1439	1.0 (0.6–1.8)	15	1079	1.3 (0.8–2.2)
Medium [>6–28]	15	2182	0.7 (0.4–1.3)	15	2203	0.8 (0.4–1.3)
High [>28–224.75]	13	1566	1.1 (0.6–2.1)	14	1893	0.9 (0.5–1.6)
			P trend = 0.76			P trend = 0.63
**Fumigant**						
**Methyl bromide** **(methyl halide fumigant)**						
None	425	45265	1.0 (ref)	425	45265	1.0 (ref)
Low [≤8]	37	2060	2.0 (1.4–2.9)	26	1680	1.8 (1.2–2.7)
Medium [>8–28]	24	3011	0.9 (0.6–1.4)	25	2501	1.1 (0.7–1.8)
High [>28–387.5]	17	2768	0.6 (0.4–1.0)	25	3571	0.8 (0.5–1.2)
			**P trend = 0.04**			P trend = 0.10

1 During the period from enrollment (1993–1997) to December 31, 2010 in NC and December 31, 2011 in Iowa.

2 Numbers of cases in columns do not sum to total number of NHL cases (n = 523) due to missing data. In the enrollment questionnaire, lifetime-days & intensity weighted life-time days of pesticide use was obtained for the insecticides: carbofuran, chlorpyrifos, coumaphos, DDVP, fonofos, permethrin and terbufos; the fungicides: captan, chlothalonil and the fumigant: methyl bromide. In the take home questionnaire lifetime-days & intensity weighted life-time days of pesticide use were obtained for the insecticides: aldicarb, carbaryl, diazinon, malathion, parathion, and phorate, the chlorinated insecticides: aldrin, chlordane, DDT, dieldrin, heptachlor, lindane, and toxaphene, the fungicides: benomyl, maneb/mancozeb and metalaxyl, therefore, numbers of NHL cases can vary among pesticides listed in the table.

3 Adjusted RR: age (<45, 45–49, 50–54, 55–59, 60–64, 65–69, ≥70), State (NC vs. IA), Race (White vs. Black), AHS herbicides (tertiles of total herbicide use-days). Statistically significant P trends are bolded.

4 Permethrin for animal use and crop use were combined into one category.

5 The distribution of life-time days of chlordane exposure was clumped into two exposed groups those who with, ≤8.75 life-time days of exposure and those with >8.75 life-time days of exposure.

We also evaluated pesticides by NHL sub-type. In the ever/never analyses (Table 2), permethrin was significantly associated with multiple myeloma, RR = 2.2 (1.4–3.5) and also demonstrated an exposure-response trend (RR = 1.0 (ref), 1.4 (0.8–2.7), 3.1 (1.5–6.2); p trend = 0.002) (Table 4). Similarly, there was an elevated risk of SLL/CLL/MCL with terbufos in ever/never analyses RR = 1.4 (0.97–2.0) and an exposure response trend (RR = 1.0 (ref), 1.3 (0.8–2.0), 1.6 (1.0–2.5); p trend = 0.05). For follicular lymphoma, lindane showed an elevated but non-significant association for ever use, RR = 1.7 (0.96–3.2) and a significant exposure-response association (RR = 1.0 (ref), 4.9 (1.9–12.6), 3.6 (1.4–9.5); p trend = 0.04). There were also two chemicals with evidence of exposure-response that were not associated with specific subtypes in the ever/never analyses: DDT (Dichlorodiphenyltrichloroethane) with SLL/CLL/MCL (RR = 1.0 (ref), 1.0 (0.5–1.8), 2.6 (1.3–4.8; p trend = 0.04); and diazinon with follicular lymphoma (RR = 1.0 (ref), 2.2 (0.9–5.4), 3.8 (1.2–11.4); p trend = 0.02) (Table 4).

**Table 4:** Pesticide exposure (Lifetime-Days of Exposure) and adjusted risks for NHL Subtypes.

Insecticides											
	SLL, CLL, MCL		Diffuse Large B-cell		Follicular B-cell		Other B-cell types		Multiple Myeloma		
	RR^3^^,^ 4(95% CI)	N^2^	RR^3.4^(95% CI)	N^2^	RR^3^^,^ 4(95% CI)	N^2^	RR^3^^,^ 4(95% CI)	N^2^	RR^3^^,^ 4(95% CI)	N^2^	NHL subtype
											Homo-geneity
											Test
											(p-value)
**Carbaryl**											
None	1.0 (ref)	42	1.0 (ref)	29	1.0 (ref)	11	1.0 (ref)	14	1.0 (ref)	22	
Low	1.1 (0.6–2.2)	19	0.8 (0.4–1.6)	17	1.6 (0.6–3.9)	10	1.8 (0.7–4.3)	10	0.7 (0.3–1.4)	14	
High	0.6 (0.3–1.3)	15	1.3 (0.6–2.8)	15	2.8 (1.0–7.4)	10	0.4 (0.1–1.5)	3	1.1 (0.7–1.8)	13	
	p trend = 0.16		p trend = 0.33		p trend = 0.06		p trend = 0.63		p trend = 0.98		0.19
**Carbofuran**											
None	1.0 (ref)	87	1.0 (ref)	78	1.0 (ref)	39	1.0 (ref)	33	1.0 (ref)	56	
Low	1.1 (0.7–1.8)	28	0.9 (0.5–1.7)	13	1.3 (0.7–2.4)	15	0.8 (0.4–1.8)	8	1.9 ((1.1–3.3)	16	
High	1.5 (0.9–2.5)	19	0.8 (0.5–1.3)	13	0.4 (0.1–1.4)	3	0.7 (0.2–2.0)	4	0.9 (0.4–1.6)	12	
	p trend = 0.16		p trend = 0.37		p trend = 0.31		p trend = 0.46		p trend = 0.57		0.52
**Chlorpyrifos**											
None	1.0 (ref)	84	1.0 (ref)	70	1.0 (ref)	33	1.0 (ref)	31	1 (ref)	58	
Low	1.2 (0.8–1.8)	31	0.9 (0.6–1.5)	22	1.6 (0.9–2.9)	20	1.2 (0.6–2.2)	14	1.0 (0.6–1.8)	17	
High	0.9 (0.6–1.3)	30	1.1 (0.6–1.7)	22	1.0 (0.5–2.1)	11	0.5 (0.2–1.3)	7	0.7 (0.4–1.3)	14	
	p trend = 0.45		p trend = 0.80		p trend = 0.94		p trend = 0.13		p trend = 0.27		0.90
**Coumaphos**											
None	1.0 (ref)	120	1.0 (ref)	92	1.0 (ref)	48	1.0 (ref)	40	1.0 (ref)	78	
Low	1.1 (0.5–2.2)	8	0.7 (0.3–1.9)	4	2.1 (0.7–5.8)	4	xxx-	4	0.7 (0.2–2.2)	3	
High	1.5 (0.6–3.4)	6	1.6 (0.6–4.5)	4	1.4 (0.5–4.0)	4	xxx-	1	1.2 (0.4–4.0)	3	
	p trend = 0.35		p trend = 0.42		p trend = 0.47		p trend = xxx		p trend = 0.84		0.63
**Diazinon**											
None	1.0 (ref)	53	1.0 (ref)	40	1.0 (ref)	15	1.0 (ref)	20	1.0 (ref)	41	
Low	1.4 (0.7–2.7)	14	1.5 (0.7–3.2)	9	2.2 (0.9–5.4)	8	xxx	3	0.4 (0.1–1.2)	4	
High	1.9 (0.98–3.6)	12	1.1 (0.5–2.4)	8	3.8 (1.2–11.4)	7	xxx	2	0.5 (0.2–1.7)	3	
	p trend = 0.06		p trend = 0.72		**p trend = 0.02**		p trend = xxx		p trend = 0.35		0.09
**DDVP**											
None	1.0 (ref)	124	1.0 (ref)	93	1.0 (ref)	48	1.0 (ref)	39	1.0 (ref)	73	
Low	0.8 (0.4–1.9)	6	1.1 (0.4–2.7)	5	1.5 (0.6–3.9)	5	1.1 (0.4–3.7)	3	2.7 (1.2–5.8)	7	
High	0.7 (0.3–1.7)	6	0.9 (0.4–2.3)	5	1.0 (0.3–3.4)	3	0.9 (0.3–3.1)	3	1.0 (0.3–2.7)	4	
	p trend = 0.49		p trend = 0.87		p trend = 0.90		p trend = 0.91		p trend = 0.81		0.96
**Fonofos**											
None	1.0 (ref)	100	1.0 (ref)	81	1.0 (ref)	45	1.0 (ref)	30	1.0 (ref)	66	
Low	1.2 (0.7–2.0)	20	1.2 (0.7–2.2)	13	1.5 (0.8–3.0)	11	1.4 (0.6–3.1)	8	1.2 (0.6–2.5)	9	
High	1.0 (0.6–1.8)	15	1.2 (0.6–2.3)	11	0.3 (0.1–1.2)	2	1.1 (0.4–2.7)	6	1.4 (0.7–3.0)	9	
	p trend = 0.96		p trend = 0.65		p trend = 0.19		p trend = 0.84		p trend = 0.33		0.35
**Malathion**											
None	1.0 (ref)	27	1.0 (ref)	20	1.0 (ref)	6	1.0 (ref)	11	1.0 (ref)	17	
Low	0.7 (0.4–1.3)	29	0.96 (0.5–1.8)	23	1.0 (0.4–2.9)	12	1.0 (0.5–2.4)	11	1.0 (0.5–2.1)	18	
High	1.0 (0.6–1.8)	22	1.0 (0.5–2.0)	20	1.6 (0.6–4.4)	11	0.3 (0.1–0.8)	6	1.0 (0.5–2.0)	17	
Ever/Never	1.0 (0.7–1.4)		0.9 (0.6–1.4)		1.3 (0.7–2.4)		0.6 (0.3–1.0)		0.9 (0.6–1.5)		
	p trend = 0.65		p trend = 0.88		p trend = 0.25		p trend = 0.17		p trend = 0.86		0.33
**Permethrin**											
None	1.0 (ref)	108	1.0 (ref)	89	1.0 (ref)	41	1.0 (ref)	38	1.0 (ref)	64	
Low	1.1 (0.6–2.0)	15	0.6 (0.3–1.2)	8	1.3 (0.6–2.7)	8	0.9 (0.3–2.7)	5	1.4 (0.8–2.7)	13	
High	0.8 (0.5–1.5)	15	1.0 (0.5–2.1)	8	1.0 (0.5–2.4)	8	0.5 (0.2–1.7)	4	3.1 (1.5–6.2)	12	
	p trend = 0.53		p trend = 0.99		p trend = 0.88		p trend = 0.28		**p trend = 0.002**		0.10
**Phorate**											
None	1.0 (ref)	48	1.0 (ref)	37	1.0 (ref)	20	1.0 (ref)	16	1.0 (ref)	36	
Low	1.0 (0.6–1.9)	14	1.4 (0.7–2.7)	15	1.1 (0.4–3.0)	5	0.9 (0.3–2.2)	6	0.7 (0.3–1.8)	6	
High	0.8 (0.4–1.6)	11	0.7 (0.3–2.1)	4	0.8 (0.3–2.2)	5	1.1 (0.4–3.5)	4	0.8 (0.3–2.4)	4	
	p trend = 0.51		p trend = 0.80		p trend = 0.67		p trend = 0.91		p trend = 0.73		0.77
**Terbufos**											
None	1.0 (ref)	72	1.0 (ref)	63	1.0 (ref)	31	1.0 (ref)	19	1.0 (ref)	59	
Low	1.3 (0.8–2.0)	32	1.2 (0.8–1.9)	29	1.6 (0.9–3.1)	15	1.8 (0.9–3.6)	17	1.1 (0.6–1.9)	12	
High	1.6 (1.0–2.5)	31	1.0 (0.5–2.0)	12	0.8 (0.4–1.7)	10	1.6 (0.7–3.9)	8	1.3 (0.7–2.7)	5	
	**p trend = 0.05**		p trend = 0.90		p trend = 0.48		p trend = 0.29		p trend = 0.42		0.63
**Chlorinated Insecticides**											
**Aldrin**											
None	1.0 (ref)	53	1.0 (ref)	46	1.0 (ref)	22	1.0 (ref)	20	1.0 (ref)	34	
Low	1.0 (0.5–2.0)	11	xxx	2	1.2 (0.4–3.8)	4	0.4 (0.1–1.5)	3	2.1 (0.9–4.7)	8	
High	1.0 (0.5–2.0)	10	xxx	3	0.8 (0.3–2.5)	4	1.1 (0.3–3.9)	3	1.2 (0.5–3.2)	6	
	p trend = 0.70		p trend = xxx		p trend = 0.21		p trend = 0.67		p trend = 0.40		0.98
**Chlordane**											
None	1.0 (ref)	48	1.0 (ref)	42	1.0 (ref)	20	1.0 (ref)	21	1.0 (ref)	32	
Low	1.8 (1.0–3.1)	16	1.0 (0.5–2.2)	8	1.7 (0.7–4.3)	6	xxx	2	1.7 (0.9–3.3)	13	
High	1.5 (0.7–3.3)	8	1.4 (0.6–3.3)	7	1.3 (0.4–4.6)	3	xxx	2	0.7 (0.2–2.2)	3	
	p trend = 0.34		p trend = 0.69		p trend = 0.70		p trend = xxx		p trend = 0.57		0.85
**DDT**											
None	1.0 (ref)	42	1.0 (ref)	34	1.0 (ref)	17	1.0 (ref)	16	1.0 (ref)	28	
Low	1.0 (0.5–1.8)	16	1.6 (0.4–3.1)	2	3.3 (1.4–8.1)	9	0.4 (0.3–2.5))	5	1.2 (0.6–2.6)	10	
High	2.6 (1.3–4.8)	15	1.4 (0.6–3.5)	3	1.1 (0.3–3.6)	4	2.1 (0.7–6.5)	5	0.8 (0.4–1.8)	9	
	**p trend = 0.04**		P trend = 0.17		p trend = 0.80		p trend = 0.64		p trend = 0.37		0.44
**Heptachlor**											
None	1.0 (ref)	58	1.0 (ref)	47	1.0 (ref)	24	1.0 (ref)	21	1.0 (ref)	40	
Low	1.1 (0.5–2.3)	9	xxx	3	xxx	2	xxx	3	1.3 (0.4–3.8)	4	
High	1.4 (0.7–3.0)	9	xxx	1	xxx	1	xxx	2	1.2 (0.4–3.6)	4	
	p trend = 0.16		p trend = xxx		p trend = xxx		p trend = xxx		p trend = 0.91		0.68
**Lindane**											
None	1.0 (ref)	57	1.0 (ref)	49	1.0 (ref)	16	1.0 (ref)	21	1.0 (ref)	43	
Low	1.2 (0.6–2.5)	10	0.6 (0.2–1.7)	4	4.9 (1.9–12.6)	6	xxx	2	xxx	3	
High	2.6 (1.2–5.6)	9	2.0 (0.6–6.5)	3	3.6 (1.4–9.5)	6	xxx	1	xxx	2	
	p trend = 0.13		p trend = 0.96		**p trend = 0.04**		p trend = xxx		p trend = xxx		0.54
**Toxaphene**											
None	1.0 (ref)	68	1.0 (ref)	47	1 (ref)	23	1.0 (ref)	22	1.0 (ref)	40	
Low	0.9 (0.4–2.3)	5	1.3 (0.5–3.3)	5	xxx	2	xxx	3	0.7 (0.2–2.0)	4	
High	0.4 (0.1–1.6)	2	0.9 (0.3–3.0)	3	xxx	2	xxx	2	0.7 (0.2–2.9)	2	
	p trend = 0.08		p trend = 0.77		p trend = xxx		p trend = xxx		p trend = 0.64		0.34
**Fungicides**											
**Captan**											
None	1.0 (ref)	118	1.0 (ref)	91	1.0 (ref)	52	1.0 (ref)	39	1.0 (ref)	76	
Low	0.9 (0.4–1.9)	7	1.1 (0.5–2.4)	7	xxx	2	xxx	3	1.4 (0.5–3.4)	5	
High	1.1 (0.5–2.6)	7	0.7 (0.1–3.1)	4	xxx	1	xxx	2	1.2 (0.5–2.9)	5	
	p trend = 0.78		p trend = 0.58		p trend = xxx		p trend = xxx		p trend = 0.75		0.92
**Chlorothalonil**											
None	1.0 (ref)	135	1.0 (ref)	107	1.0 (ref)	60	1.0 (ref)	50	1.0 (ref)	84	
Low	0.9 (0.4–2.3)	5	1.1 (0.4–3.1)	4	xxx	3	−xxx	1	1.1 (0.4–2.8)	5	
High	1.1 (0.4–3.3)	4	0.3 (0.1–1.2)	2	xxx	2	−xxx	1	0.7 (0.6–2.3)	3	
	p trend = 0.83		p trend = 0.09		p trend = xxx		p trend = xxx		p trend = 0.56		0.76
**Metalaxyl**											
None	1.0 (ref)	60	1.0 (ref)	45	1.0 (ref)	25	1.0 (ref)	23	1.0 (ref)	39	
Low	2.8 (1.4–5.8)	9	1.1 (0.4–2.6)	7	xxx	3	−xxx	2	0.4 (0.1–1.1)	4	
High	1.1 (0.4–2.8)	6	1.0 (0.4–2.7)	5	xxx	2	−xxx	1	1.1 (0.4–3.2)	4	
	p trend = 0.99		p trend = 0.97		p trend = xxx		p trend = xxx		p trend = 0.87		0.92
**Maneb/Mancozeb**											
None	1.0 (ref)	69	1.0 (ref)	49	1.0 (ref)	25	1.0 (ref)	26	1.0 (ref)	41	
Low	2.1 (0.7–6.0)	4	4.0 (1.4–11.6)	4	xxx	2	−xxx	0	1.0 (0.4–2.5)	5	
High	1.2 (0.3–4.0)	3	0.9 (0.3–3.1)	3	−xxx	1	−xxx	0	2.2 (0.5–9.5)	2	
	p trend = 0.84		p trend = 0.74		p trend = xxx		p trend = xxx		p trend = 0.28		0.82
**Fumigant**											
**Methyl Bromide**											
None	1.0 (ref)	126	1.0 (ref)	86	1.0 (ref)	58	1.0 (ref)	44	1.0 (ref)	76	
Low	1.1 (0.5–2.2)	9	4.0 (2.2–7.4)	15	1.4 (0.5–4.2)	4	3.6 (1.3–9.8)	5	1.0 (0.5–2.1)	8	
High	0.8 (0.4–1.8)	8	1.0 (0.5–2.1)	11	0.3 (0.1–1.1)	3	1.3 (0.3–5.0)	3	0.8 (0.4–1.8)	8	
	p trend = 0.58		p trend = 0.67		p trend = 0.08		p trend = 0.56		p trend = 0.63		0.59

1 During the period from enrollment (1993–1997) to December 31, 2010 in NC and December 31, 2011 in Iowa.

2 Numbers of cases in columns do not sum to total number of NHL cases (n = 523) due to missing data. Ever/never use of all 26 pesticides (table 3) do not always match with exposure-response data in table 4 because of missing data to calculate lifetime-days of use.

3 Adjusted for age (<45, 45–49, 50–54, 55–59, 60–64, 65–69, ≥70), State (NC vs. IA), Race (White vs. Black), AHS herbicides (in tertiles of total herbicide use-days). Significant RR and 95% confidence limits are bolded.

4 RR was not calculated if the number of exposed cases for any NHL subtype was <6 and these cells are marked XXX. Four pesticides included in Table 2 (i.e., aldicarb, benomyl, dieldrin and parathion) were not included in Table 4 because no NHL subtype included≥6 cases of a specific cell types with lifetime-days of exposure.

The pattern of increased CLL/SLL/MCL risk with increased use of DDT and terbufos remained after both insecticides were placed in our model concurrently. CLL/SLL/MCL risk increased with DDT use (RR = 1.0 (ref), 0.9 (0.5–4.7); 2.4 (1.1–4.7); p trend = 0.04), and a pattern of increased CLL/SLL/MCL risk was also observed with terbufos use (RR = 1.0 (ref), 1.1 (0.6–2.1), 1.7 (0.9–3.3) p trend = 0.07), although the trend was not significant for terbufos. Similarly, the pattern of increased follicular lymphoma risk with lindane use and diazinon use remained after both insecticides were placed in our model concurrently. Follicular lymphoma risk increased with diazinon use (RR = 1.0 (ref), 4.1 (1.5–11.1); 2.5 (0.9–7.2); p trend = 0.09), and a similarly, pattern of increased follicular lymphoma risk was observed with lindane use (RR = 1.0 (ref), 1.6 (0.6–4.1), 2.6 (0.8–8.3) p trend = 0.09), although neither remained statistically significant (Table 4).

Three chemicals showed elevated risks in ever/never analyses for certain subtypes, with no apparent pattern in exposure-response analyses: metalaxyl and chlordane with SLL/CLL/MCL, RR = 1.6 (1.0–2.5) and RR = 1.4 (0.97–2.0) respectively, and methyl bromide with diffuse large B-cell lymphoma RR = 1.9 (1.1–3.3). Although there was evidence of association by subtype, and polytomous logit models indicated homogeneity across subtypes for lindane (p = 0.54), DDT (p = 0.44) and any other pesticide evaluated in this study (e.g., permethrin (p = 0.10), diazinon (p = 0.09), terbufos (p = 0.63), (last column in Table 4).

There was no evidence of confounding of the total NHL associations with either lindane or DDT. We also calculated RR for those who completed both the phase 1 enrollment and take-home questionnaires and the phase 2 questionnaire (n = 17,545) and found no meaningful difference in the RR that also included imputed exposures, although there was an increase in precision of risk estimates (i.e., narrower confidence intervals) when we included phase 2 imputed data (n = 54,306) (data not shown). Lagging exposures by five years did not meaningfully change the association between lindane or DDT and total NHL (data not shown). The significant exposure-response trends linking use of a particular pesticide to NHL and certain NHL subtypes did not always correspond to a significant excess risk among those who ever used the same pesticide. For chemicals for which the detailed information was only asked about in the take-home questionnaire, we evaluated potential differences between the ever/never analyses based on the enrolment questionnaire and data from the same sub-set of participants who completed the exposure-response in the take-home questionnaire and found no meaningful differences in the results. We also evaluated the impact of using an updated definition of NHL; when using the original ICD-O-3 definition of NHL^19^, lifetime-days of lindane use remained significantly associated with NHL risk (RR = 1.0 (ref), 1.3 (0.7–2.6), 1.2 (0.6–2.8), 2.7 (1.3–5.4), p trend = 0.006). The trend between total NHL and lifetime-days of DDT, however, was less clear and not statistically significant (RR = 1.0 (ref) 1.3 (0.9–1.8), 1.1 (0.5–2.1), 1.4 (0.8–2.6), p trend = 0.32) [Table S3 in File S1]. Carbaryl and diazinon showed non-significant trends with the older definition of NHL, but not with the newer definition used here.

## Discussion

A significant exposure–response trend for total NHL was observed with increasing lifetime-days of use for two organochlorine insecticides, lindane and DDT, although RRs from ever/never comparisons were not elevated. On the other hand, terbufos use showed a significant excess risk with total NHL in ever vs. never exposed analysis, but displayed no clear exposure-response trend. Several pesticides showed significant exposure-response trends with specific NHL subtypes however, when polytomous models were used to test the difference in parametric estimates of trend among the five NHL subtypes, there was no evidence of heterogeneity in the sub-types for specific chemicals. The subtype relationships that looked particularly interesting were DDT and terbufos with the SLL/CLL/MCL subtype, lindane and diazinon with the follicular subtype, and permethrin with MM. These pesticide-NHL links should be evaluated in future studies.

Lindane (gamma-hexachlorocyclohexane) is a chlorinated hydrocarbon insecticide. Production of lindane was terminated in the United States in 1976, but imported lindane was used to treat scabies and lice infestation and for agricultural seed treatment [21] until its registration was cancelled in 2009 [22], the same year production was banned worldwide [23]. In our study, 3,410 people reporting ever using lindane (6%) prior to enrollment, 433 reported use at the phase 2 questionnaire (1%), indicating that use had dropped substantially. Oral administration of lindane has increased the incidence of liver tumors in mice and less clearly, thyroid tumors in rats [24]. Lindane produces free radicals and oxidative stress (reactive oxygen species [ROS]) [25] and has been linked with chromosomal aberrations in human peripheral lymphocytes in vitro [26].

Lindane has been linked with NHL in previous epidemiologic studies. A significant association between lindane use and NHL was observed in a pooled analysis of three population-based case-control studies conducted in the Midwestern US, with stronger relative risks observed for greater duration and intensity of use [27]. NHL was also associated with lindane use in a Canadian case-control study [28]. Lindane was significantly associated with NHL risk in an earlier report from the AHS [29]. We are not aware of any previous study that assessed the association between a NHL subtype and lindane use. The exposure-response pattern with total NHL and the follicular lymphoma subtype indicates a need for further evaluation of lindane and NHL.

DDT is an organochlorine insecticide that was used with great success to control malaria and typhus during and after World War II [29] and was widely used for crop and livestock pest control in the United States from the mid-1940s to the 1960s [30]. Its registration for crop use was cancelled in the US in 1972 [30] and banned worldwide for agricultural use in 2009, but continues to be used for disease vector control in some parts of the world [23]. In our study, 12,471 participants (23%) reported ever using DDT prior to enrollment; 12%, 8.7% and 2.3% responding to the take-home questionnaire reported their first use occurred prior to the 1960s, during the 1960s, and during the 1970s, respectively. The National Toxicology Program classifies DDT as “reasonably anticipated to be a human carcinogen” [31] and IARC classifies DDT as a “possible human carcinogen (2B)” [12], both classifications were based on experimental studies in which excess liver tumors were observed in two rodent species. Epidemiology data on the carcinogenic risk of DDT is inconsistent. NHL was not associated with use of DDT in a pooled analysis of three case-control studies in the U.S. where information on exposure was obtained from farmers by questionnaire [32]. There also was no association between the use of DDT and NHL in our study when we used an earlier definition of NHL [18], suggesting some of the inconsistency may be due to disease definition. In the large Epilymph study, no meaningful links between DDT and the risk of NHL, or diffuse large B cell lymphoma were observed, and only limited support was found for a link to CLL [33], although a case-control study of farmers in Italy suggested increased risk of NHL and CLL with DDT exposure [34]. NHL was not associated with serum levels of DDT in a prospective cohort study from the U.S. [35], but NHL was associated with the DDT-metabolite p, p’-DDE, as well as chlordane and heptachlor-related compounds (oxychlordane, heptachlor epoxide) and dieldrin, in a study with exposure measured in human adipose tissue samples [36]. In a Danish cohort, a higher risk of NHL was associated with higher prediagnostic adipose levels of DDT, cis-nonachlor, and oxychlordane [37]. In a Canadian study, analytes from six insecticides/insecticide metabolites (beta-hexachlorocyclohexane, p, p’-dichloro-DDE, hexachlorobenzene (HCB), mirex, oxychlordane and transnonachlor) were linked with a significant increased risk with NHL [38]. However, in an analysis of plasma samples from a case-control study in France, Germany and Spain, the risk of NHL did not increase with plasma levels of hexachlorobenzene, beta-hexachlorobenzene or DDE [39]. In this analysis, NHL was significantly associated with reported use of DDT, but not with the other organochlorine insecticides studied (i.e., aldrin, chlordane, dieldrin, heptachlor, toxaphene). Our findings add further support for an association between DDT and total NHL and our results on SLL/CLL/MCL are novel and should be further explored.

Permethrin is a broad-spectrum synthetic pyrethroid pesticide widely used in agriculture and in home and garden use as an insecticide and acaricide, as an insect repellant, and as a treatment to eradicate parasites such as head lice or mites responsible for scabies [40]. This synthetic pyrethroid was first registered for use in the United States in 1979 [40]. The U.S. Environmental Protection Agency classified permethrin as “likely to be carcinogenic to humans” largely based on the observed increase incidence of benign lung tumors in female mice, liver tumors in rats and liver tumors in male and female mice [41]. Permethrin was not associated with NHL overall in our study, nor in pooled case-control studies of NHL from the U.S (the NHL definition in use at the time of the study did not include MM) [42]. In our analysis, however, the risk of MM increased significantly with lifetime-days of exposure to permethrin, as had been noted in an earlier analysis of AHS data [43]. We are unaware of other studies that have found this association.

Terbufos is an organophosphate insecticide and nematicide first registered in 1974 [44]. The EPA classifies terbufos as Group E, i.e., “Evidence of Non-Carcinogenicity for Humans” [44]. We found some evidence for an association between terbufos use and NHL, particularly for the SLL/CLL/MCL subtype. NHL was not associated with terbufos in the pooled case-control studies from the U.S. [42] but there was a non-significant association between terbufos and small cell lymphocytic lymphoma [10].

Diazinon is an organophosphate insecticide registered for a variety of uses on plants and animals in agriculture [45]. It was commonly used in household insecticide products until the EPA phased out all residential product registrations for diazinon in December 2004 [45.46]. In an earlier evaluation of diazinon in the AHS, a significant exposure-response association was observed for leukemia risk with lifetime exposure-days [47]. While there was no link between diazinon and NHL overall in this analysis, there was a statistically significant exposure-response association between diazinon and the follicular lymphoma subtype and an association with the SLL/CLL/MCL subtype that was not statistically significant. Diazinon was previously associated with NHL in pooled case-control studies from the U.S. and particularly with SLL [10].

Several other insecticides, fungicides and fumigants cited in recent reviews of the pesticide-cancer literature suggested etiological associations with total NHL [8], [9], these include: oxychlordane, trans-nonachlor, and cis-nonachlor which are metabolites of chlordane; and dieldrin and toxaphene among NHL cases with t(14,18) translocations. We did not find a significant association between chlordane and total NHL nor with any NHL subtype, but we did not have information about chlordane metabolites to make a more direct comparison. Similarly we did not observe a significant association between dieldrin nor toxaphene and total NHL nor with any NHL subtypes. Mirex (1,3-cyclopentadiene), an insecticide, and hexachlorobenzene, a fungicide, were also associated with NHL risk [8], [9] but we did not examine these compounds in the AHS.

This study has a number of strengths. It is a large population of farmers and commercial pesticide applicators who can provide reliable information regarding their pesticide use history [48]. Information on pesticide use and application practices was obtained prior to onset of cancer. An algorithm that incorporated several exposure determinants which predicted urinary pesticide levels was used to develop an intensity-weighted exposure metric in our study [20]. Exposure was ascertained prior to diagnosis of disease, which should eliminate the possibility of case-response bias [14]. Because of the detailed information available on pesticide use, we were able to assess the impact for the use of multiple pesticides. For example, we evaluated total pesticide use-days, and specific pesticides found to be associated with NHL or its subtypes in the AHS. We found no meaningful change in the associations with DDT, lindane, permethrin, diazinon and terbufos from such adjustments. Information on many potential NHL risk factors was available and could be controlled in the analysis.

Most epidemiological investigations of NHL prior to 2007 [17] did not include CLL and MM as part of the definition. These two subtypes made up 37% (193/523) of the NHL cases in this analysis. This is a strength of our study in that the definition of NHL used here is based on the most recent classification system [16], [17] and will be relevant for comparisons with future studies. On the other hand, the inclusion of MM and CLL in the recent definition of NHL makes comparisons of our findings with earlier literature challenging, because the NHL subtypes may have different etiologies. For example, DDT was not significantly associated with NHL using the older definition, but was significantly associated with the NHL using the most recent definition of NHL because of its association with the SLL/CLL/MCL subtype (Table S1 in File S1). On the other hand, carbaryl and diazinon were associated with the old definition of NHL (although non-significantly) but not with the new definition. Lindane, however, was associated with both definitions of NHL. Lindane was significantly associated with the follicular lymphoma subtype and this subtype was included in the older and newer definition of NHL. No other pesticides were significantly associated with NHL under the old definition (Table S3 in File S1).

Although this is a large prospective study, limitations should be acknowledged. A small number of cases exposed to some specific pesticides could lead to false positive or negative findings. We also had reduced statistical power to evaluate some pesticides for total days of use and intensity-weighted days of use because some participants did not complete the phase one take-home questionnaire and the tests of homogeneity between specific pesticides and specific NHL subtypes were underpowered. Some chance associations could occur because of multiple testing, i.e., a number of pesticides, several NHL subtypes, and more than one exposure metric. Despite the generally high quality of the information on pesticide use provided by AHS participants [48], [50], misclassification of pesticide exposures can occur and can have a sizeable impact on estimates of relative risk, which in a prospective cohort design would tend to produce false negative results [49].

### Conclusion

Our results showed pesticides from different chemical and functional classes were associated with an excess risk of NHL and NHL subtypes, but not all members of any single class of pesticides were associated with an elevated risk of NHL or NHL subtypes, nor were all chemicals of a class included on our questionnaire. Significant pesticide associations were between total NHL and reported use of lindane and DDT. Links between DDT and terbufos and SLL/CLL/MCL, lindane and diazinon and follicular lymphoma, and permethrin and MM, although based on relatively small numbers of exposed cases, deserve further evaluation. The epidemiologic literature on NHL and these pesticides is inconsistent and although the findings from this large, prospective cohort add important information, additional studies that focus on NHL and its subtypes and specific pesticides are needed. The findings from this large, prospective cohort add important new information regarding the involvement of pesticides in the development of NHL. It provides additional information regarding specific pesticides and NHL overall and some new leads regarding possible links with NHL subtypes that deserve evaluation in future studies.

## Supporting Information

File S1 **This file contains Table S1, Table S2, and Table S3.** Table S1, Frequency of NHL in Agricultural Health Study applicators using New (Interlymph hierarchical classification of lymphoid neoplasms) and Older Definitions (ICD-O-3). Table S2, Pesticides included in the Agricultural Health Study questionnaires by Chemical/Functional Class. Table S3, Pesticide exposure (lifetime-days) and adjusted risks of total NHL incidence (Older definition [ICD-O-3]).
(DOC)
