# Supplementary material for: Non-Hodgkin Lymphoma Risk and Insecticide, Fungicide and Fumigant Use in the Agricultural Health Study
Source: PLoS One. 2014 Oct 22;9(10):e109332. doi: 10.1371/journal.pone.0109332 (PMC4206281; doi:10.1371/journal.pone.0109332)
Supplement: File S1 — This file contains Table S1, Table S2, and Table S3. Table S1, Frequency of NHL in Agricultural Health Study applicators using New (Interlymph hierarchical classification of lymphoid neoplasms) and Older Definitions (ICD-O-3). Table S2, Pesticides included in the Agricultural Health Study questionnaires by Chemical/Functional Class. Table S3, Pesticide exposure (lifetime-days) and adjusted risks of total NHL incidence (Older definition [ICD-O-3]). (DOC) [file pone.0109332.s001.doc]

**File SI (Supporting Information File)**

**Supporting Information Legend**

**Table S1. Frequency of NHL in Agricultural Health Study applicators using New (Interlymph hierarchical classification of lymphoid neoplasms) and Older Definitions (ICD-O-3)**

**Table S2. Pesticides included in the Agricultural Health Study questionnaires by Chemical/Functional Class**

**Table S3. Pesticide exposure (lifetime-days)^1^ and adjusted risks of total NHL incidence^2^ (Older definition [ICD-O-3])^3^**

**Table S1. Frequency of NHL in Agricultural Health Study applicators using New (Interlymph hierarchical classification of lymphoid neoplasms) and Older Definitions (ICD-O-3)**

| **Lymphoma subtype and type**  **(ICD-O-3 codes)^1^** | **Number NHL cases, ICD-O-3 new definition**  **(InterLymph hierarchical classification)^1^** | **Number cases NHL, original ICD-O-3 definition^2^** | **NHL_LYMPH SEER Recode^1^** |
| --- | --- | --- | --- |
| **SLL/CLL/MCL (Mature NHL, B-cell)** |  |  |  |
| Small lymphocytic lymphoma (9670) | 32 | 32 | 08 |
| Chronic lymphocytic leukemia  (9823) | 95 | 0 | 08 |
| Mantle -cell lymphoma (9673) | 21 | 21 | 10 |
| **Diffuse Large B-cell Lymphoma (Mature NHL, B-cell)** |  |  |  |
| DLBCL (9680) | 117 | 117 | 13 |
| **Follicular Lymphoma (Mature NHL, B-cell)** |  |  |  |
| Follicular lymphoma (9690, 9691,9695,9698) | 67 | 67 | 21 |
| **Other B-cell Types** |  |  |  |
| Precursor acute lymphoblastic leukemia/lymphoma  (9835(B), 9836) | 4 | 0 | 07 |
| Waldenstrom macroglobulinemia (9761) | 6 | 0 | 12 |
| Lymphoplasmacytic lymphoma (9671) | 3 | 3 | 11 |
| Hairy-cell leukemia (9940) | 6 | 0 | 22 |
| NHL, NOS (9591(B), 9675(B)) | 12 | 12 | 26 |
| Burkitt lymphoma/leukemia (9687) | 1 | 1 | 17 |
| Extranodal marginal zone lymphoma (MZL), Malt type & Nodal MZL (9699) | 14 | 14 | 19, 20 |
| Plasma cell neoplasms  Plasmacytoma (9734, 9731) | 7 | 0 | 23 |
| **Multiple myeloma (9732)** | 97 | 0 | 24 |
| **Other NHL Types** |  |  |  |
| Precursor acute lymphoblastic leukemia/lymphoma  (9835(T), 9837) | 1 | 0 | 27 |
| Mycosis fungoides (9700) | 8 | 8 | 28 |
| Peripheral T-cell lymphoma, NOS (9702) | 3 | 3 | 30 |
| Anaplastic large cell lymphoma, T or null cell (9714) | 2 | 2 | 33 |
| Enteropathy type T-cell lymphoma (9717) | 1 | 1 | 35 |
| Primary cutaneous anaplastic large cell lymphoma (9718) | 1 | 1 | 37 |
| T-cell large granular lymphocytic NK leukemia (9831) | 1 | 0 | 40 |
| NHL, NOS (9591(T)) | 1 | 1 | 42 |
| Precursor acute lymphoblastic leukemia/lymphoma  (9727(U), 9835(U)) | 3 | 1 | 43 |
| NHL, NOS (9591(U), 9675(U)) | 7 | 7 | 45 |
| Lymphoid neoplasm, NOS (9590,9820[U]) | 12 | 11 | 47 |
| T-cell lymph, nasal-type/aggressive NK leukemia (9719) | 1 | 1 | 39 |
| Total | 523 | 303 |  |

Lineage: B=B-cell, T=T-cell, U=Unknown

^1^ <http://seer.cancer.gov/lymphomarecode> based on Morton LM et al. Blood, 2007;110:695-708.

^2^ Percy C. et al., SEER, NCI: 2001.

**Table S2. Pesticides included in the Agricultural Health Study questionnaires by Chemical/Functional Class**

| **Chemical/functional class** | **Pesticide** |
| --- | --- |
| Acetamide herbicide | Metolachlor^1^, alachlor^1^ |
| Carbamate herbicide | Butylate^2^, S-ethyl dipropylthiocarbamate (EPTC)^1^ |
| Other herbicides | Chlorimuron ethyl^2^, 2,4-dichlorophenoxyacetic acid (2,4-D)^1^, dicamba^1^, glyphosate^1^, petroleum oil^2^, imazethapyr^1^, paraquat^2^, pendimethalin^2^, 2,4,5-trichlorophenoxyacetic acid (2,4,5-T)^2^, silvex (2,4,5-TP)^2^, trifluralin^1^ |
| Triazine/triazinone herbicides | Atrazine^1^, cyanazine^1^, metribuzin^2^ |
| Carbamate insecticides | Carbofuran^1^, aldicarb^2^, carbaryl^2^ |
| Chlorinated insecticides | Aldrin^2^, chlordane^2^, dichlorodiphenyltrichloroethane (DDT)^2^, dieldrin^2^ heptachlor^2^, lindane^2^, toxaphene^2^ |
| Organophosphate insecticides | Chlorpyrifos^1^, coumaphos^1^, diazinon^2^, dichlorvos^1^, fonofos^1^, malathion^2^, parathion (ethyl or methyl)^2^ phorate^2^, terbufos^1^ |
| Other insecticides | Permethrin (crops & animals)^1^, trichlorfon^1^ |
| Fungicides | Benomyl^2^, chlorothalonil ^1^, captan^1^, maneb/mancozeb^2^, metalaxyl^2^, ziram^2^ |
| Fumigants | Methyl bromide^2^, aluminum phosphide ^2^ ,ethylene dibromide^2^, carbon tetrachloride/carbon disulfide^2^ |
| ^1^Full exposure information in enrolment questionnaire (22 pesticides)  ^2^Full exposure information in take-home questionnaire (28 pesticides) | |

**Table S3**

**Pesticide exposure (lifetime-days)^1^ and adjusted risks of total NHL incidence^2^ (Older definition [ICD-O-3])^3^**

|  | | | | |
| --- | --- | --- | --- | --- |
|  | Older definition | | Newer Definition | |
| **Pesticide** | NHL cases | RR^4,5^(95% CI) by Total Days of Exposure | NHL cases | RR^4,5^(95% CI) by Total Days of Exposure |
| **Aldicarb**  **(carbamate-insecticide)** |  |  |  |  |
| None | 123 | 1.0 (ref) | 238 | 1.0 (ref) |
| Low [<8.75] | 4 | 0.9(0.3-2.9) | 7 | 1.1(0.5-2.3) |
| Medium [>8.75-25.5] | 1 | 1.0(0.5-2.8) | 5 | 0.9(0.3-2.5) |
| High [>25.5] | 2 | 1.5(0.8-3.0) | 5 | 0.5 (0.2-1.3) |
|  |  | P trend=0.31 |  | P trend=0.23 |
| **Carbofuran**  **(carbamate-insecticide)** |  |  |  |  |
| None | 168 | 1.0 (ref) | 317 | 1.0 (ref) |
| Low [<8.75] | 29 | 1.1(0.7-1.6) | 63 | 1.2 (0.9-1.6) |
| Medium [>8.75-38.75] | 20 | 1.0(0.6-1.6) | 32 | 0.8(0.6-1.2) |
| High [>38.75] | 23 | 0.9(0.6-1.5) | 44 | 0.97 (0.7-1.4) |
| P trend |  | P trend=0.73 |  | P trend=0.69 |
| **Carbaryl**  **(carbamate-insecticide)** |  |  |  |  |
| None | 67 | 1.0 (ref) | 128 | 1.0 (ref) |
| Low [<8.75] | 24 | 0.8(0.5-1.4) | 54 | 1.1 (0.7-1.6) |
| Medium [8.75-56] | 26 | 1.2(0.7-2.0) | 43 | 0.9 (0.6-1.2) |
| High [>56] | 21 | 1.5(0.8-2.6) | 39 | 1.0(0.7-1.6) |
|  |  | P trend=0.12 |  | P trend=0.87 |
| **Chlorpyrifos**  **(organophosphate-insecticide)** |  |  |  |  |
| None | 159 | 1.0 (ref) | 300 | 1.0 (ref) |
| Low [<8.75] | 39 | 1.1(0.8-1.6) | 71 | 1.1 (0.9-1.5) |
| Medium [>8.75-44] | 47 | 1.0(0.7-1.4) | 65 | 1.1(0.8-1.4) |
| High [>44] | 28 | 0.9(0.6-1.4) | 67 | 0.8(0.6-1.1) |
|  |  | P trend=0.71 |  | P trend=0.11 |
| **Coumaphos**  **( organophosphate-insecticide)** |  |  |  |  |
| None | 215 | 1.0(ref) | 411 | 1.0(ref) |
| Low [<8.75] | 9 | 1.1(0.6-2.1) | 16 | 1.0 (0.6-1.7) |
| Medium [>8.75-38.75] | 8 | 1.1(0.5-2.1) | 14 | 1.2 (0.7-2.1) |
| High [>38.75] | 7 | 1.6(0.7-3.4) | 13 | 1.2(0.7-2.0) |
|  |  | P for trend=0.24 |  | P for trend=0.50 |
| **DDVP**  **(dimethyl phosphate-insecticide)** |  |  |  |  |
| None | 219 | 1.0 (ref) | 407 | 1.0 (ref) |
| Low [<8.75] | 10 | 0.8(0.4-1.5) | 19 | 1.4(0.9-2.1) |
| Medium [>8.75-87.5] | 8 | 2.3(1.1-4.7) | 17 | 1.2(0.7-1.9) |
| High [>87.5] | 7 | 0.7(0.3-1.5) | 17 | 0.9(0.6-1.5) |
|  |  | P trend=0.54 |  | P trend=0.78 |
| **Diazinon**  **(organophosphorous-insecticide)** |  |  |  |  |
| None | 91 | 1.0 (ref) | 187 | 1.0 (ref) |
| Low [<8.75] | 15 | 1.3(0.8-2.3) | 28 | 1.1(0.7-1.6) |
| Medium [>8.75-25] | 14 | 1.2(0.5-2.8) | 19 | 1.0(0.6-1.8) |
| High [>25} | 12 | 1.5(0.8-3.0) | 23 | 1.2(0.7-1.9) |
|  |  | P trend=0.19 |  | P trend=0.52 |
| **Fonofos**  **(organophosphorous-insecticide)** |  |  |  |  |
| None | 189 | 1.0 (ref) | 349 | 1.0 (ref) |
| Low [<20] | 23 | 1.2(0.8-1.8) | 47 | 1.3(0.96-1.8) |
| Medium [>20-50.75] | 15 | 1.0(0.6-1.7) | 28 | 1.1(0.7-1.6) |
| High [>50.75] | 18 | 0.9(0.5-1.5) | 37 | 1.1 (0.7-1.5) |
|  |  | P trend=0.64 |  | P trend=0.83 |
| **Malathion**  **(organophosphorous-insecticide)** |  |  |  |  |
| None | 48 | 1.0 (ref) | 90 | 1.0 (ref) |
| Low [<8.75] | 38 | 0.9(0.6-1.4) | 75 | 0.97 (0.7-1.3) |
| Medium [>8.75-38.75] | 22 | 0.6(0.4-1.0) | 47 | 0.7(0.5-1.1) |
| High [>38.75] | 29 | 0.9(0.6-1.5) | 57 | 0.9 (0.6-1.3) |
|  |  | P trend=0.95 |  | P trend=0.63 |
| **Parathion (ethyl or methyl)**  **(organophosphorous insecticide** |  |  |  |  |
| None | 120 | 1.0(ref) | 228 | 1.0(ref) |
| Low [<8.75] | 3 | 0.7(0.2-2.2) | 9 | 1.0(0.5-2.0) |
| Medium [> 8.75-24.5] | 3 | 1.1(0.3-3.5) | 6 | 1.4(0.6-3.2) |
| High [>.24.5] | 2 | 0.7(0.2-2.7) | 6 | 0.8(0.3-1.8) |
|  |  | P trend=0.59 |  | P trend=0.64 |
| **Permethrin** |  |  |  |  |
| None | 198 | 1.0(ref) | 371 | 1.0 (ref) |
| Low | 19 | 0.9 (0.6-1.5) | 38 | 1.1(0.8-1.5) |
| Medium | 16 | 1.0(0.6-1.8) | 31 | 0.8(0.5-1.2) |
| High | 17 | 0.7(0.4-1.2) | 33 | 1.2(0.8-1.7) |
| P trend |  | P trend=0.25 |  | P trend=0.54 |
| **Phorate** |  |  |  |  |
| None | 87 | 1.0 (ref) | 171 | 1.0 (ref) |
| Low | 15 | 0.9(0.5-1.5) | 27 | 0.8(0.5-1.2) |
| Medium | 20 | 1.6(0.9-2.6) | 33 | 1.4(0.96-2.1) |
| High | 6 | 0.4(0.2-0.9) | 18 | 0.6(0.4-1.1) |
|  |  | P trend =0.47 |  | P trend =0.25 |
| **Terbufos** |  |  |  |  |
| None | 136 | 1.0 (ref) | 267 | 1.0 (ref) |
| Low | 43 | 1.1(0.8-1.6) | 82 | 1.2(0.9-1.5) |
| High | 32 | 1.8(1.2-2.6) | 54 | 1.6(1.2-2.1) |
| Ever/Never | 31 | 1.1 (0.7-1.6) | 57 | 1.1 (0.8-1.5) |
|  |  | P trend=0.49 |  | P trend=0.43 |
| **Chlorinated Insecticides** | | |  |  |
| **Aldrin** |  |  |  |  |
| None | 103 | 1.0 (ref) | 193 | 1.0 (ref) |
| Low | 11 | 0.7(0.4-1.3) | 27 | 0.9(0.6-1.4) |
| Medium | 9 | 0.5(0.2-1.0) | 16 | 0.8(0.5-1.3) |
| High | 6 | 1.2(0.5-2.9) | 17 | 0.9(0.5--1.5) |
|  |  | P trend=0.93 |  | P trend=0.58 |
| **Chlordane** |  |  |  |  |
| None | 96 | 1.0 (ref) | 179 | 1.0 (ref) |
| Low | 18 | 1.3(0.6-1.7) | 47 | 1.3(0.97-1.9) |
| Medium | 5 | 2.0(0.8-5.0) | 0 | xxx |
| High | 7 | 0.8(0.4-1.8) | 23 | 1.1(0.7-1.7) |
|  |  | P trend=0.84 |  | P trend=0.43 |
| **Dieldrin** |  |  |  |  |
| None | 121 | 1.0 (ref) | 235 | 1.0 (ref) |
| Low | 3 | 0.6(0.2-1.9) | 7 | 0.7(0.3-1.5) |
| Medium | 3 | 1.6(0.5-5.1) | 8 | 2.3(1.1-4.7) |
| High | 1 | 0.7(0.1-5.0) | 2 | 0.7(0.2-2.9) |
|  |  | P trend=0,80 |  | P trend=0.47 |
| **DDT** |  |  |  |  |
| None | 82 | 1.0 (ref) | 152 | 1.0 (ref) |
| Low | 21 | 1.3(0.9-1.8) | 43 | 1.3(0.9-1.8) |
| Medium | 10 | 1.1(0.5-2.1) | 28 | 1.1(0.7-1.7) |
| High | 15 | 1.4 (0.8-2.6) | 27 | 1.7 (1.1-2.6) |
|  |  | P trend=0.32 |  | P trend=0.02 |
| **Heptachlor** |  |  |  |  |
| None | 104 | 1.0 (ref) | 205 | 1.0 (ref) |
| Low | 11 | 1.0(0.5-1.9) | 21 | 1.0(0.6-1.6) |
| Medium | 6 | 0.9(0.4-2.1)) | 18 | 1.5 (0.9-2.4) |
| High | 5 | 0.9(0.3-3.2.1) | 7 | 0.7 (0.3-1.4) |
|  |  | P trend=0.76 |  | P trend=0.82 |
| **Lindane** |  |  |  |  |
| None | 98 | 1.0 (ref) | 205 | 1.0 (ref) |
| Low | 11 | 1.3(0.7-2.6) | 18 | 1.2(0.7-1.9) |
| Medium | 10 | 1.2(0.6-2.8) | 13 | 1.0(0.6-1.7) |
| High | 9 | 2.7(1.3-5.4) | 14 | 2.5(1.4-4.4) |
|  |  | P trend=0.006 |  | P trend=0.004 |
| **Toxaphene** |  |  |  |  |
| None | 109 | 1.0 (ref) | 214 | 1.0 (ref) |
| Low | 6 | 0.7(0.3-1.7) | 14 | 0.8(0.5-1.4) |
| Medium | 7 | 1.8(0.8-4.0) | 13 | 1.5(0.9-2.7) |
| High | 3 | 0.7(0.2-2.2) | 6 | 0.6(0.3-1.4) |
|  |  | P trend=0.91 |  | P trend=0.50 |
| **Fungicides** | | |  |  |
| **Captan** |  |  |  |  |
| None | 218 | 1.0 (ref) | 407 | 1.0 (ref) |
| Low | 8 | 0.8(0.4-1.5) | 15 | 0.9(0.6-1.5) |
| Medium | 8 | 1.4(0.7-3.0) | 16 | 1.2(0.7-2.2) |
| High | 5 | 0.5(0.2-1.5) | 14 | 0.8(0.5-1.5) |
|  |  | P trend=0.36 |  | P trend=0.52 |
| **Chlorothalonil** |  |  |  |  |
| None | 262 | 1.0 (ref) | 474 | 1.0 (ref) |
| Low | 5 | 1.3(0.5-3.2) | 13 | 0.6(0.3-1.2) |
| Medium | 4 | 0.5(0.2-1.5) | 9 | 0.9(0.5-1.7) |
| High | 4 | 0.4(0.1-1.1) | 9 | 0.8(0.4-1.6) |
|  |  | P trend= 0.08 |  | PP trend= 0.52 |
| **Metalaxyl** |  |  |  |  |
| None | 111 | 1.0 (ref) | 209 | 1.0 (ref) |
| Low | 11 | 0.8(0.4-1.5) | 16 | 1.6(0.8-3.3) |
| Medium | 3 | 0.7(0.2-2.2) | 15 | 1.3(0.6-2.6) |
| High | 5 | 0.9(0.3-2.3) | 13 | 0.9(0.4-1.9) |
|  |  | P trend=0.74 |  | P trend=0.78 |
| **Maneb/Mancozeb** |  |  |  |  |
| None | 116 | 1.0 (ref) | 228 | 1.0(ref) |
| Low | 4 | 2.4(0.9-6.7) | 8 | 1.3(0.8-2.2) |
| Medium | 4 | 1.1(0.3-3.2) | 9 | 0.8(0.4-1.3) |
| High | 3 | 1.0(0.3-3.4) | 7 | 0.9(0.5-1.6) |
|  |  | P trend=0.99 |  | P trend=0.63 |
| **Fumigants** | | | | |
| **Methyl bromide**  **(methyl halide fumigant)** |  |  |  |  |
| None | 227 | 1.0 (ref) | 425 | 1.0(ref) |
| Low [<8] | 16 | 2.5(1.5-4.3) | 37 | 1.8(1.2-2.7) |
| Medium [>8-28] | 15 | 1.4(0.8-2.5) | 24 | 1.1(0.7-1.8) |
| High [>28] | 15 | 0.7(0.4-1.3) | 17 | 0.8(0.5-1.2) |
|  |  | P=0.15 |  | P trend=0.10 |

^1^ During the period from enrollment (1993-1997) to December 31, 2010 in NC and December 31, 2011 in Iowa.

^2^ Adjusted RR: age(<45, 45-49, 50-54, 55-59, 60-64, 65-69, >70), State (NC vs. IA), Race (White vs. Black), AHS herbicides (tertiles of total herbicide use-days)

^3^ Percy C. et al., SEER, NCI: 2001.

^4^  Permethrin for animal use and crop use were combined into one category.

^5^ The distribution of life-time days of chlordane exposure was clumped into two exposed groups those who with, <8.75 life-time days of exposure and those with >8.75 life-time days of exposure.
